# The Connection between Diseases of the Eye and Diseases of the Teeth

**Published:** 1898-03

**Authors:** Charles Stedman Bull

**Affiliations:** New York


### International Dental Journal.

Vol. XIX. March, 1898. No. 3.

### Original Communications.[1]

### THE CONNECTION BETWEEN DISEASES OF THE EYE AND DISEASES OF THE TEETH.[2]

BY CHARLES STEDMAN BULL, A.M., M.D., OF NEW YORK.

The relation which is supposed to exist between affections of the teeth and diseases of the eye has been familiar to us all for many years, and the belief in its existence is almost as old as the science of medicine itself. The difficulty has always been to explain exactly what this connection is, and how the diseased process is propagated from the mouth to the eye, or, in the reverse direction, from the eyes to the mouth.

Attempts have been made to classify the lesions of the eye which are supposed to be of dental origin by dividing them into two classes,—viz., those lesions which are of reflex origin and those which are of an inflammatory nature. All such attempts, however, have been generally too vague and too positive, too schematic; for a reflex lesion may later become an inflammatory lesion, and must thus come to be regarded as the irritative phase of an inflammatory phenomenon. In these latter days of what may be called the "microbic craze" we are, perhaps, too prone to consider the so-

[1] The editor and publishers are not responsible for the views of authors of papers published in this department, nor for any claim to novelty, or otherwise, that may be made by them. No papers will be received for this department that have appeared in any other journal published in the country.

[2] Read before The New York Institute of Stomatology, January 4, 1898.

called reflex lesions as more likely to be of microbic origin, thus restricting the reflex ocular phenomena within still narrower limits.

Attempts have been made in the past by the advocates of the reflex theory to regard all lesions of the anterior segment of the eyeball, in cases of dental disease, as due to some disturbance of the trifacial nerve; while lesions of the posterior part of the eyeball are considered to be of an inflammatory, infectious nature, propagated directly from the diseased teeth or upper jaw to the orbit, and thence to the eyeball. This is a fanciful classification which is not justified by our clinical or pathological knowledge.

It is probable that lachrymation, due to irritation of the terminal filaments of the trifacial nerve, is of reflex origin, as may be also amblyopia with peripheral narrowing of the visual field. In the latter case, however, it is possible that the irritation may be propagated by inflammation along the optic canal, where the optic nerve may be compressed or inflamed by the septic elements of inflammation, infected, and thus give rise to defects of vision and a narrowing of the visual field.

It is very possible that a dental lesion causing a sensitive impression in the trifacial may reach the great sympathetic nerve, or the ciliospinal centre, or even the bulbar enlargement of the cord, and give the impulse to a greater or less number of neurons, according to the degree of impressionability of the subject or for other reasons. Any or all of these reasons would suffice for the retention of the sensitive impressions in several nuclei and the production of varying motor or vasomotor phenomena.

Congestive phenomena of reflex origin, by means of irritation of the filaments of the trifacial, are said to cause dilatation of the vessels of the eyeball, and even to set up keratitis and iritis. This, however, is by no means certain, and is more than doubtful. It would seem more rational to believe, for example, in a case of empyema of the maxillary antrum due to dental caries, followed by iritis, that an infectious osteoperiostitis had ensued, with metastasis of the pus through the medium of the blood-vessels and lymphatics, to the tissue of the iris and choroid. This method of propagation has not yet been proved to occur, but it is highly probable. It is not yet positively known that infectious microbes coming from a suppurating focus must always set up the same suppurative inflammation in a new tissue when carried there by the vessels and lymphatics. Cases of iritis occurring in the course of abscess of the maxillary antrum with dental caries are probably of pure microbic origin. Mere conditions of irritation and congestion of the eye may be the starting-point of a microbic infection.

We know that infection of dental origin may extend to the sinuses of the face, not only the antrum, but the frontal sinus, ethmoid sinus, or sphenoid antrum, by the natural opening into each of these sinuses. Infectious inflammation of the maxillary antrum, consecutive to an alveolar dental periostitis, frequently extends to the orbit, and these cases of cellulitis of the orbit are due in most instances to periostitis of the floor of the orbit extending from the antrum. The pus may extend to the orbit by stripping the periosteum from below upward, or, more frequently, through the medium of the communicating veins. The veins of the maxillary sinus and of the periosteum of the upper jaw open generally into the ophthalmo-facial vein, which, coming from the pituitary membrane, passes through the sphenopalatine foramen, anastomoses with the intraorbital veins, and empties into the facial vein below the level of the malar bone. Inflammation of this vein, of microbic origin, might of itself cause a thrombophlebitis of the cavernous sinus. The inflammatory process might also follow the course of the lymphatics. Thus alveolar dental periostitis may cause thrombophlebitis of the cavernous sinus and secondary orbital phlebitis, not only when starting in the superior maxilla but also when confined to the inferior maxilla.

An interesting fact has been brought out by Reynier and Parinaud. They have proved that in certain cases of orbital cellulitis from alveolar dental periostitis and abscess of the antrum, the pus does not reach the orbit by stripping up the periosteum, for there is no periosteum. The pus spreads through the bone itself. Pus starting in an alveolus may perforate it; but, on the other hand, it may not destroy the alveolar wall, but pass through the canaliculi or foramina at the apex of the alveolus, and thence into the infraorbital notch, or through one of several orifices situated in front of the lachrymal sac, in the ascending ramus of the superior maxilla. These orifices often communicate directly with the nasal fossæ or antrum, so that the pus coming from a carious alveolus may reach the orbit either by passing through the sinus or by avoiding it.

In children the maxillary sinus is much smaller than in the adult, and does not bear the same relations to the orbit. It is effaced in part by the development of the alveoli of the first and second dentition. The relation of these alveoli vary with the age of the subject; but in some cases the alveolus of second dentition may extend as far as the orbital arch. In the case of the canine tooth, the alveoli of the first and second dentition, more or less exactly superimposed, the one on the other, often communicate with each other by an orifice, which gives passage to both nerve and vessels, and later, by the absorption of the wall of separation, the one resulting alveolus ascends as far as the orbit.

In both children and adults the infectious focus may remain localized, or it may spread by various channels more or less rapidly and gain progressively the sinuses of the face and base of the skull, through the veins of the optic foramen or vault of the orbit.

It would seem wiser to give up any attempt to classify the ophthalmic lesions met with in connection with morbid processes in the teeth, and to consider the subject under two heads: *First*, lesions of the eye met with in the course of primary and secondary dentition. *Second*, lesions of the eye occurring in the course of abnormal diseased processes in the teeth.

*First*. Lesions of the eye met with in the course of dentition, both primary or early and secondary: The connection existing between certain affections of the eye and early dentition is very close, and is recognized by all practising physicians. Some of the ocular manifestations met with in the course of early eruption of the first teeth are sympathetic in nature and probably reflex in origin, such as photophobia and lachrymation. Others are of a distinctly inflammatory nature, though also reflex in origin, such as the various forms of ulceration of the cornea and conjunctiva. Catarrhal conjunctivitis, phlyctenular conjunctivitis and keratitis, ulcers of the cornea, lagophthalmos from spasm of the levator of the upper lid, marginal blepharitis, mydriasis, and a peculiar form of cataract are affections of the eyes not infrequently met with during early dentition. Many authors have cited such instances. Sous, in the *Journal de Médecine de Bordeaux*, 1896, reports an obstinate case of keratitis during early dentition. Auge, in 1881, published a paper on blepharoconjunctivitis during teething. Foucher, in 1895, published cases of herpetic conjunctivitis; as have also Tavignot, in 1871, Galezowski, in 1888, Chevalier and Décaisne, in 1869, and, more recently, Knies, in 1895. The case reported by Sous is interesting. A child was attacked by diffuse keratitis of the right eye, and a cure followed lancing of the gums. In the following year the left eye became inflamed in the same way, and the process involved not only the cornea but the sclera also. A tooth was removed to allow a deviated tooth to take its normal position, and in three days the cornea had resumed its normal transparency and all signs of inflammation vanished. All these so-called superficial inflammations of the eye are generally considered to be reflex, and are relieved and generally cured by incisions in the swollen gums. I have under my care at present a child, aged ten months, in excellent physical condition, with good digestion, who for nearly three months has had a series of phlyctenular ulcerations on the margin of the cornea in both eyes, appearing in groups, and accompanied by great photophobia and considerable general conjunctivitis, and very pronounced swelling and engorgement of the gums. Local treatment has proved of little use, but on every occasion after lancing of the gums there has been an immediate improvement in the photophobia and the ulcers began to heal. The teeth have pushed themselves forward very rapidly, and with each new attack of swelling of the gums a fresh crop of small ulcers have appeared on the margin of the cornea.

One of the most interesting ocular lesions met with in connection with difficult early dentition is the zonular cataract. Horner was one of the first to call attention to this in 1864, and he supposed it to be due to the influence of infantile rickets. Arlt first noticed the coincidence of infantile convulsions and zonular cataract, and thought that in the convulsions met with during dentition some solution of due physiological contact took place between the denser central portion and the more fluid peripherical portion of the lens, and that this mechanical lesion manifested itself as a stationary zonular or lamellar opacity. Horner noticed that these patients showed cranial deformities and malformation of the first teeth, which he attributed to rickets. Out of sixty-five cases of zonular cataract, forty-eight had infantile convulsions, and in thirty-six cases there were dental and cranial deformities. The teeth are thick and coarse, the incisors are cubical, and the enamel terminates abruptly on the neck of the tooth in a swollen ridge. The horizontal furrows in the enamel become visible to the naked eye, and near the cutting edge a horizontal row of round holes marks the position of one of these excavated grooves. The body of the tooth terminates in a convex border at the cutting edge. Sometimes the enamel is wanting in the grooves and the discolored dentine shows through. Story, in 1886, reported nine cases. He does not believe that rickets is always the cause. The dental defect is due to an arrest of development. The period of the growth of the enamel is of limited duration, somewhere between the sixth and the ninth month. He thinks that there is a close similarity between the lens and the dental enamel in origin and development, and that it is, therefore, natural that they should be affected by the same pathological agents. Both are formed by an involution of the epithelium from the surface of the embryo, and in both the growth proceeds from the deeper layer of this epithelium, when separated from the rest of the epiblast by the interposition of a mesoblastic layer. There is this difference, however: the enamel organ has only a brief functional existence and then disappears, but the growth of the lens proceeds during many years.

Hutchinson published a paper in the *Lancet* in 1875, in which he stated that these imperfect teeth were, as a rule, met with only in connection with the zonular cataract of early childhood. The latter was always symmetrical and quite stationary, and was not associated with any particular diathesis. The defective development of the teeth was seen in the incisors, canines, and first molars, the latter being the test-teeth. The bicuspids usually escape. In contradistinction to Story and Horner, he believes that the permanent teeth are alone affected. The incisors and canines are pitted, dirty, and rough, with spinous edges. The non-development of the enamel and the erosion of the exposed dentine are the essential features. With these teeth and the zonular cataract there is usually a history of infantile convulsions.

Some have held that these dental defects usually result from inflammation of the gums in infancy, and that mercury is the chief cause, the mercury having been administered during the period when the enamel was undergoing calcification.

Hutchinson believes that the connection between zonular cataract and convulsions is universal. Weiss (1876) holds the same views. The general testimony seems to be in favor of the view that when zonular cataract is found with a history of convulsions, the permanent teeth will certainly be malformed. The distinct characteristic of a genuine "convulsion tooth," unaffected by congenital syphilis, is the shortness of its enamel, the dentine projecting beyond the enamel edge and being more or less honey-combed.

*Second.* Lesions of the eye occurring in the course of diseased processes in the permanent teeth: The reflex theory has many strong advocates, one of the most recent being Knies (1895) and from the time of Mackenzie, in 1833, down to the present day most writers on ophthalmic diseases have given more or less adhesion to the doctrine. It is only when we reach the period of bacteriological investigations that the reflex theory of origin begins materially to lose ground.

Leber (Graefe und Saemisch, *Handbuch der gesammten Augenheilkunde*, vol. v., 1877) reports numerous observations of the occurrence of visual disturbance through irritation of the extraocular branches of the trigeminus in diseases of the teeth, such as simple dental caries with severe continuous toothache and sensitiveness of the tooth to pressure, or suppuration in the alveoli with prolongation of the inflammation to the antrum. The teeth involved are generally in the upper jaw, and usually the molars. The eye symptoms are photophobia, retinal hyperæsthesia, photopsia, loss of accommodation, and, more rarely, amblyopia and amaurosis. All these symptoms simulate sympathetic troubles. The ophthalmoscopic evidence may be negative, though occasionally there are signs of retinitis and optic neuritis.

Desmarres (1858) believed that there was a real pathological connection between diseases of the eye and diseases of the teeth, and that the connection might be either direct by continuity of tissue or reflex through the nervous system. The lesions on the side of the eye might be either organic or functional.

Nuel (Wecker et Landolt, "Traité Complet des Maladies des Yeux," vol. iii., 1887) is even more positive in his statements. Generally the superior dental nerves are involved. If amblyopia is really present, one or more molars in the upper jaw will be found sensitive. If the superior dental nerves are involved, there will always be conjunctival and ciliary injection, with ciliary neuralgia, lachrymation, photophobia, and sometimes blepharospasm and spasm of accommodation. Nuel considers these symptoms all reflex: reflex vasomotor dilatation in the conjunctival and ciliary vessels, reflex contraction of the muscles of the face, and reflex innervation of the lachrymal gland. If amblyopia exist, there is usually also narrowing of the visual field, and the vision becomes worse with each fresh attack of dental neuralgia.

Galezowski (1872) is an advocate of the reflex theory, but believes that the reflex influence is exerted not in the brain but in the ganglion or node of Gasse, and works in both ways, from the teeth to the eyes and *vice versa*.

Without quoting the names or opinions of the long list of authors who have expressed their views on the subject, it seems to be definitely settled that a relation exists between certain lesions of the eye and certain diseased conditions of the teeth. Knies formulates the modern views as follows:

1. Conjunctivitis of the phlyctenular variety is known to be intimately connected with teething, even with the second dentition.

2. It is undoubtedly true that keratitis, iritis, glaucoma, muscular paralysis, asthenopia, amblyopia without visible lesion, supraorbital neuralgia, and exophthalmos with and without orbital cellulitis, are caused directly or indirectly by carious teeth.

3. When paralysis of accommodation appears during an attack of toothache it probably results simply from the lack of vigorous innervation on account of the distressing pain.

4. Muscular insufficiency or paralysis, with diplopia, may also be explained as paresis due to enfeebled innervation.

5. Spasm of accommodation and nictitation are both reflex symptoms frequently observed with toothache, as is also neuralgia of one or more of the branches of the trifacial nerve.

6. Amblyopia and amaurosis are by no means infrequent complications of carious teeth. Any positive ophthalmoscopic evidence should lead us to assume a common cause for the dental pain and the visual disturbance.

7. A spasm of the levator muscle of the upper lid leading to the appearance known as lagophthalmos has not infrequently been described in connection with pain in carious teeth.

8. Orbital cellulitis with development of abscess in the lower lid has been noted in caries of the teeth of the upper jaw with the development of abscesses round the diseased teeth.

9. Exophthalmos, or protrusion of the eye, may be caused by serous infiltration of the orbital tissue directly connected with disease of the alveoli.

10. Conversely, pain in the teeth of the upper jaw is by no means an infrequent symptom in iritis and cyclitis with severe neuralgic pain in the branches of the trifacial nerve; and toothache has been unmistakably recognized as one of the prodromal signs of glaucoma.

For the sake of convenience we may perhaps divide the lesions of the eye met with in connection with lesions of the teeth into three groups, as follows:

1. Those dependent on vasomotor disturbances, which would include all cases of disturbance of nutrition, all inflammatory cases, and the reflex cases, such as amblyopia and amaurosis without ophthalmoscopic evidence.

2. Those dependent on disturbances of sensibility, such as ciliary neuralgia and the various forms of asthenopia.

3. Those characterized by disturbances of motility, including all cases of spasm and paralysis of the muscles of the eye, including the iris and ciliary muscle.

The eye-complications of dental disease are of varied nature. Beginning with the most superficial structures of the eye, the first subject that will engage our attention is the *eyelids.*

Herpetic eruption of the lids in the course of the terminal branches of the fifth nerve is often met with in connection with carious teeth, and resists all treatment until the teeth are extracted or properly filled.

Hutchinson has reported an interesting case of constant spasmodic contraction of the levator of the lid in a lady who had four decayed molar teeth in both jaws on the corresponding side. These were removed, and all ciliary neuralgia at once ceased, but the spasm remained. The left upper first molar contained an amalgam stopping, which, on being removed, revealed an exposure of the pulp. This tooth was also removed, and improvement in the muscular spasm began at once, and it shortly stopped altogether. This may be regarded as purely reflex.

On the other hand, Redard has reported a case of paralysis of the levator of the lid which resisted all treatment. A careful examination of the mouth showed extensive caries of the upper third molar, and, on this being removed, the paralysis began to improve, and in a few days the lid had completely regained its power.

Ely has reported a case of paresis of the orbicularis muscle associated with spasm of the ciliary muscle in a patient with an abscess at the root of the third upper molar on the right side. This tooth was extracted, and the ocular symptoms immediately disappeared.

When we come to consider the lesions of *cornea* and *sclera*, the number of cases reported in connection with diseased teeth is almost numberless. Ulcers of the cornea, superficial and interstitial keratitis, and scleritis are repeatedly mentioned. Marginal phlyctenular keratitis is a very common accompaniment of early and second dentition as well as of carious teeth in the adult. Ely reports a typical case in a man aged thirty-five, who had severe neuralgia of the right side of the face, with ulcer of the conjunctiva and scleritis of the right eye. There was in the right upper canine tooth an ulcerated spot just below the gum, and the nerve was exposed. The nerve was destroyed, the ulcerated spot treated, and the neuralgia ceased at once, and in a few days the eye became perfectly well. Hern has also reported a number of cases of scleritis of undoubted dental origin. *Iritis* has also been observed in connection with interstitial keratitis in cases of carious teeth, and the teeth being properly cared for, the iritis promptly got well.

The *loss* of *accommodation* due to *paralysis* of the *ciliary muscle* has received special attention from several authors.

Schmidt-Rimpler (Graefe und Saemisch, *Handbuch der gesammten Augenheilkunde*, vii. p. 72, 1877) gives the results of his experience in ninety-two cases, and draws the following conclusions:

1. In consequence of the pathological irritation of the dental branches of the trifacial nerve there may result more or less marked limitation of the range of accommodation.

2. These limitations of accommodation may be either unilateral or bilateral. When unilateral, they always appear in the eye on the affected side.

3. These limitations of accommodation occur most frequently in the young, while at an advanced age they occur rarely or never.

4. They are due to an intraocular increase of tension, proceeding from a reflex irritation of the vasomotor nerves.

In seventy-three of the ninety-two cases reported, the accommodation was diminished far below the normal standard. In most of the cases the accommodation was regained after the removal of the trouble in the teeth, either by the cure of the carious process or by the extraction of the diseased teeth. The degree of limitation of the accommodation varied greatly.

Schmidt-Rimpler believes that the irritation of the branches of the trigeminus due to carious teeth causes in a reflex manner an irritation of the vasomotor nerves of the eye, and that irritation of these vasomotor nerves produces an increase of the intraocular tension, which diminishes the accommodation.

Ely (*New York Medical Record*, March 11, 1882) reports in detail the case of a man, aged thirty-one, who had not only paralysis of accommodation in the right eye, but also paralysis of the right internal rectus muscle. The root of the first upper molar tooth on the right side was denuded, rough, and sensitive. The nerve of the tooth was dead, the alveolar process was absorbed, and there was extensive suppuration in the adjacent parts. The condition of the tooth was rectified, and the symptoms all disappeared.

Galezowski (*Progrès Médicale*, No. 29, 1888) has an article in which he draws similar conclusions to those of Schmidt-Rimpler.

Dunn (*American Journal of Ophthalmology*, October, 1891) reports a case of paralysis of accommodation, with impaired vision and severe ciliary neuralgia in a patient, who was found to have an abscess at the root of the second molar tooth in the lower jaw on the left side. The tooth was extracted and the alveolar cavity antiseptically treated, and in a few days all the ocular symptoms entirely disappeared.

When we come to consider the subject of amblyopia and amaurosis, whether reflex or not, in connection with the existence of carious teeth or lesions in the jaw, the list of authors becomes very large. From the date of publication of Mackenzie's "Treatise on Diseases of the Eye," in 1833, to the last edition of Swanzy's "Hand-Book of Diseases of the Eye," in 1897, scarcely a text-book has appeared which does not treat more or less distinctly of the subject. Hocken in 1842 published a treatise on amaurosis, in which a number of cases are reported as due to carious teeth. Most of the cases reported have been associated with dental neuralgia of the molars of the upper jaw, and the lesion has been caries, sometimes complicated by abscesses of greater or less extent at the roots of the teeth. It should be understood that all cases of amblyopia or amaurosis considered under this head have been those in which no ophthalmoscopic evidence of disease has ever been noticed from the beginning to the end of the trouble. There is usually lachrymation, photophobia, conjunctival or ciliary injection, and occasionally spasm of accommodation, but the fundus remains healthy. The defect of vision may be unilateral or bilateral, and when the latter exists the vision is always worse on the side corresponding to the diseased teeth. If the defect in vision is marked, the field of vision is usually concentrically narrowed, and both vision and limitation of the field of vision become worse with each exacerbation of the neuralgia. The photophobia in these cases is due to the existing retinal hyperæsthesia.

Park (*Annals of Ophthalmology*, January, 1893) has reported a case of a patient who complained of headache, loss of vision in the right eye, and some photopsic manifestations. A few days later vision in the right eye was reduced to perception of light. The ophthalmoscopic examination was negative. There was concentric narrowing of the field of vision. An examination of the mouth revealed five decayed teeth in the right upper jaw, and an artificial plate was found resting and pressing on the decayed roots. The roots were all extracted, and vision began to improve at once, and in two months was completely restored.

In the *Courier Médical* for 1890 will be found the report of a case of a lady, aged thirty, who complained of failing vision in the left eye accompanied by severe toothache in the second left upper molar tooth. In one week from the onset of the attack the left eye became entirely blind, without any ophthalmoscopic evidence of disease. The diseased tooth was extracted, and pus immediately flowed from the alveolar cavity. Examination revealed a small piece of wooden toothpick, which was removed from the cavity, and in a few days there was complete restoration of vision.

I have recently had under my care a lady who presented herself complaining of lachrymation, photophobia, and loss of accommodation in the right eye. In two days vision in this eye began to fail, and the field of vision became concentrically narrowed. Repeated ophthalmoscopic examinations revealed nothing abnormal. In about a week from the onset severe neuralgia of the right side of the face set in. She was sent to her dentist, who discovered that the second molar in the right upper jaw, which had been filled, was very sensitive to pressure. The filling was removed and the nerve found inflamed. At this time the vision had been reduced to perception of light. The nerve was destroyed and the cavity was again filled. The neuralgia, lachrymation, and photophobia ceased at once, and in less than a week vision was completely restored, and the field of vision regained its normal extent.

Proceeding next in the course adopted, from without inward, from the superficial affections of the eye to the deeper and more serious lesions, in connection with diseased processes in the teeth, we come to consider *inflammation of the optic nerve*, or *optic neuritis*, including the immediately surrounding zone of the retina. Of this disease we have a number of undoubted cases on record.

In 1893, Hermann (*Centralblatt für Praktische Augenheilkunde*, December, 1893) reported a case of inflammation of the optic nerve of the variety known as papillitis, occurring in a patient in whom no cause for the lesion could be found until the teeth were examined, when a carious tooth was found accompanied by toothache. Unfortunately, the extraction of the tooth and the cure of the suppurative process had no beneficial effect upon the inflammatory process in the optic nerve.

Despagnet (*Annales d'Oculistique*, May, 1893) reports a very clear and interesting case occurring in a woman, aged twenty-four, in whom there was optic neuritis with dilatation of the iris. An examination of the mouth showed caries of the last molar of the upper jaw on the corresponding side, with extensive periostitis of the alveolar process and a sequestrum of the alveolar arch. There was suppurative inflammation in the antrum directly connected with the diseased alveolus, and the suppurative process was found to have extended to the orbit as far back as the foramen opticum and here had involved the optic nerve.

Hirsch (*Wiener Medicinische Wochenschrift*, 1893, No. 34) reports a case of optic neuritis ending in atrophy of the nerve and blindness, in which the disease evidently started in the left second upper molar. There was an extensive abscess involving the gum, cheek, and lower lid, which had perforated externally. A probe could be passed from the external opening in the cheek along the floor and inner wall of the orbit. The infection had extended from the tooth-cavity and set up alveolar periostitis, which had involved the antrum and floor of the orbit, and set up orbital cellulitis, inflammation, and eventually atrophy of the optic nerve.

Feuer (*Annals of Ophthalmology*, 1895) reports two cases of optic neuritis ending in a complete cure and restoration of vision after extraction of the carious teeth. In the first case there were three decayed teeth with osteoperiostitis of the alveolar arch and antrum, with exophthalmos and optic neuritis. In the second case the wisdom-tooth in the right upper jaw was diseased, with periostitis of the alveolar arch and of the floor and inner wall of the orbit. The loss of sight was complete in the right eye, and coincided with the occurrence of inflammation and pain in the orbital floor. The vision was completely restored in three months.

Cases of this sort might be multiplied almost indefinitely, but enough have been presented to show the connection which actually exists between diseased teeth and the inflammation of the optic nerve.

Choroiditis, or inflammation of the choroid coat of the eye, has been mentioned by a number of authors as occasionally due to reflex irritation from a decayed tooth, and several cases have been reported. One of the most notable cases is reported by Sous in the *Journal de Médecine de Bordeaux* for 1896. The patient was a girl, aged twenty, who complained of failing vision in the right eye. An examination showed both keratitis punctata and choroiditis. The first right upper molar was carious and had been recently filled, but was still painful. Pressure on this tooth caused an acute pain in the eye. The filling was removed and vision began at once to improve, the keratitis rapidly disappeared, and within a week the choroiditis was gone. The tooth was then refilled, and on the next day there was a return of all the symptoms. The filling was again removed, and the symptoms again rapidly disappeared. Here the irritation of a dental nerve acted in a reflex manner upon the sensory ophthalmic nerves and set up a secretory choroiditis.

Anomalies of the ocular muscles in connection with dental lesions have been repeatedly reported, and the writer of this paper has seen several such cases.

Terrier (*Recueil d'Ophthalmologie*, 1876) has reported a very obstinate case of spasmodic contraction of the motor muscles of an eye and of the orbicularis muscle of the eyelids, which was entirely relieved by extraction of the carious teeth.

Hutchinson (*British Medical Journal*, December, 1895) reports a case of lagophthalmos due to dental irritation. The patient, a lady, suffered from marked spasm of the left upper eyelid. The left second and third molars in both jaws were badly decayed, and there was intense left facial neuralgia in all the branches of the fifth nerve. All four teeth were extracted, and the neuralgia ceased at once, but the spasm of the lids continued. The left first upper molar had an amalgam stopping; this was removed and the pulp found exposed. This tooth was removed, and an improvement in the muscular spasm of the lids began at once, and in six months it was entirely cured.

Ely (*New York Medical Record*, March, 1882) reports a case of a woman, aged forty, who had marked paralysis of the right third nerve or oculomotorius. Several decayed teeth were found in the right upper jaw, and the gums were badly swollen. The teeth were extracted, the alveolar cavities and gums were treated antiseptically, and the symptoms of muscular paralysis all disappeared.

The subject of periostitis of the bones of the orbit, as a consequence of periostitis of the alveolar arch due to decayed teeth, is so intimately connected with orbital cellulitis that the two will be considered together. These constitute some of the most serious cases, not only as regards danger to the integrity of the eye, but also to the life of the patient, whichever fall under the observation of the oculist. Hirsch, Péchin, Juler, Schwendt, Pagenstecher, Vossius, Burnett, Snell, Fage, and many others have all reported cases.

Schwendt published an inaugural dissertation on orbital cellulitis in 1882, in which he collected the reports of forty-four cases, of which seven were directly due to carious teeth. The cellulitis was generally preceded by abscess of the antrum, periostitis of the floor of the orbit, and sometimes by suppuration in the ethmoid, with discharge of pus from the nose.

Vossius, in 1884, reported a case of orbital cellulitis with thrombophlebitis of the orbital veins and optic neuritis, with complete restoration of vision after extraction of a carious molar. This case was probably due to a rapid infectious inflammation of the veins and lymphatics with thrombosis, through the alveolar veins into the antrum, and thence by a branch which perforates the floor of the orbit and empties into the infraorbital vein.

Snell, in 1890, reported to the London Ophthalmological Society the case of a young girl, aged fourteen, who had two decayed teeth in the upper jaw, which were extracted and periostitis found in the alveolar arch. Acute orbital cellulitis followed, and the orbital tissue was freely incised, giving exit to a large quantity of pus, but death followed in a few days from meningitis.

Fage (*Receuil d' Ophthalmologie*, 1893) reports the case of a man, aged twenty-nine, who applied for treatment. There was protrusion of the left eye with marked chemosis, œdematous lids, dilated iris, and the cheek swollen and painful. There was fever and a fetid odor to the breath. The gums of the left upper and lower jaws were swollen and there were several carious teeth. The first left upper molar was wanting, having been removed a few days before on account of severe toothache. The symptoms became rapidly worse, and the orbital tissue was freely incised, but no pus appeared. An incision in the lower lid gave exit to fetid pus. A probe passed freely through the alveolar cavity of the first molar into the antrum, and pus flowed from the latter. This treatment, together with antiseptic irrigation, brought about a rapid recovery.

Galezowski reports a somewhat similar case, occurring in a young woman, aged twenty. He comments on the rather marked frequency of alveolar abscess due to carious teeth, he having seen nine cases in two years among seventeen thousand cases of eye-disease.

There seems to be no doubt that in addition to the rather benign reflex disturbances in the eye accompanying more or less serious disease in the teeth and jaws, we also not infrequently meet with grave ocular lesions due to an infectious process, and here our anatomical knowledge enables us to follow the channel of propagation accurately. I think it may be stated that the primary evil starts in the periosteum around a carious alveolus, develops at first slowly, and if recognized and properly treated, it may be cured without an abscess. If, however, the alveolar dental periostitis extends to the antrum, the next step will be an abscess of the antrum, periostitis of its walls, including the root of the antrum or floor of the orbit, which, in its turn, sets up orbital cellulitis, ending in suppuration, with possible destruction of the eyeball from panophthalmitis, or inflammation of the optic nerve and atrophy from strangulation of the nerve by pressure.

The various steps in the destructive process here outlined are well illustrated in the report of a single case, published by Juler in 1895 (*British Medical Journal*, October 19). A boy complained of pain in one of the upper molar teeth, and this toothache was followed by pain in the jaw, then by pain in the orbit, exophthalmos, orbital cellulitis, and suppurative panophthalmitis, and total destruction of the eye. The tooth was extracted, and was found extensively diseased, with caries of the alveolar wall, periostitis, abscess at the root of the tooth communicating with an abscess of the antrum. The eye was enucleated, and the floor of the orbit was found extensively diseased, with a large hole communicating with the cavity of the antrum.

In this somewhat rapid sketch of a subject of almost equal interest to both dentist and oculist, there does not seem to be any reasonable doubt of the close connection which exists between certain diseased processes in the eyes and certain abnormal physiological and pathological conditions of the teeth. The modern ophthalmologist certainly recognizes the existence of this connection, though he perhaps has not insisted upon it sufficiently in the published literature.

